# Effectiveness of multimethod, community-based educational interventions on the knowledge and attitude to birth preparedness and complications readiness among women in southwest Nigeria

**DOI:** 10.1136/bmjph-2023-000203

**Published:** 2024-06-10

**Authors:** Ifeoma P Okafor, Mobolanle R Balogun, Adekemi O Sekoni, Duro C Dolapo, Claudia A Hawkins, Bosede B Afolabi

**Affiliations:** 1 Department of Community Health & Primary Care, University of Lagos, College of Medicine, Lagos, Nigeria; 2 Department of Community Medicine, Nile University of Nigeria, Abuja, Nigeria; 3 Institute for Global Health- Centre for Global Communicable and Emerging Infectious Diseases, Northwestern University, Chicago, Illinois, USA; 4 Department of Medicine, Northwestern University Feinberg School of Medicine, Chicago, Illinois, USA; 5 Department of Obstetrics & Gynaecology, University of Lagos, College of Medicine, Lagos, Nigeria

**Keywords:** Public Health, Community Health, Preventive Medicine, Social Medicine

## Abstract

**Background:**

Birth preparedness and complications readiness (BP/CR) is an effective strategy to reduce maternal and newborn morbidity and mortality.

**Aim:**

To assess the effect of educational interventions on women’s knowledge and attitude regarding BP/CR in southwest Nigeria.

**Methods:**

A quasi-experimental study was carried out over 1 year (May 2019–April 2020) in Lagos, southwest Nigeria. Intervention was delivered using multiple educational methods: health education sessions, information, education, and communication materials, and mHealth. A total of 2600 women were recruited by multistage sampling. Data were collected using interviewer-administered questionnaires and analysed with Epi Info and SPSS V.25 software. Summary and inferential statistics were done involving four-way analysis. The level of significance was set at p<0.05. Regression analysis applied to intervention group (IG). A 50% cut-off was used to categorise respondents into adequate and inadequate knowledge of BP/CR.

**Results:**

Mean age of the respondents: 31.2±5.4 years for the IG, 30.4±6.0 years for the control group (CG); p=0.007. Most women in both groups had formal education, were employed and had their last antenatal care in health facilities. At baseline, both groups had overall inadequate knowledge of BP/CR which improved significantly post intervention only in the IG. For the IG: 9.4% (pre), 52% (post), (p<0.001); CG: 0.2% (pre), 0.5% (post), (Fisher’s exact p=0.624). Most respondents in both groups had a positive attitude to BP/CR, the intervention had no significant influence on this in the IG (p=0.504).

Predictors of adequate knowledge of BP/CR included being of Yoruba tribe, (adjusted OR (AOR) 2.83, 95% CI 1.06 to 7.54), being employed, (AOR 1.31; 95% CI 1.04 to 5.87) and having a baby 6 months prior to the study (AOR 2.62; 95% CI 1.31 to 5.24).

**Conclusion:**

Findings have implications for the design and implementation of relevant policy and community interventions to reduce maternal mortality. Further research can examine the role of financial exclusion in inadequate knowledge.

**WHAT IS ALREADY KNOWN ON THIS TOPIC**

- Birth preparedness and complications readiness (BP/CR) is a strategy aimed at reducing maternal and newborn mortality and morbidity.
- Globally, it is an essential component of focused antenatal care package.

**WHAT THIS STUDY ADDS**

- Women had very inadequate knowledge of BPCR despite using formal maternity care services in their recent pregnancy.
- Educational intervention delivered in different methods improved their knowledge.

**HOW THIS STUDY MIGHT AFFECT RESEARCH, PRACTICE OR POLICY**

- The study provides evidence for policy interventions that strengthen health systems for quality maternity care and use of various methods at the community level to improve knowledge of BPCR. Women’s financial empowerment is equally important.

## Introduction

 The high maternal and newborn deaths recorded in many low-income and middle-income countries (LMICs) continue to be a major source of public health concern. In Nigeria, the maternal mortality ratio is 512/100 000 live births, representing one of the highest worldwide.^1^ Also, about 34 of every 1000 neonates die within 1 month of birth.^2^ For every maternal death, there are 20 or more others who suffer serious morbidities.^3^ The magnitude of these unwarranted deaths prompted their inclusion in the Millennium Development Goals over a decade ago. Many LMICs fell short of meeting the desired Maternal, Newborn and Child Health (MNCH) goals due to a myriad of challenges. One of the suggestions to remedy this was to ensure universal access to MNCH information involving the society at large and increase funding and research.^4^ Now, with the transition to Sustainable Development Goals (SDGs), countries are to do more to reduce significantly maternal, newborn and child deaths by 2030.^4^

Three-delay model theorises that events leading up to maternal, newborn and child deaths can be explained by delays in seeking care, delays in reaching care and delays in receiving adequate care once at the point of service.^5^ Birth preparedness and complications readiness (BP/CR) is a strategy to promote the timely use of skilled maternal and neonatal care thus reducing delays in seeking and obtaining this care. It is a process of preparing or planning for birth and anticipating what to do in the case of obstetric complications. BP/CR includes knowing the danger signs, identifying a skilled provider and the location of the nearest health facility (HF) with emergency obstetric care, arranging for transportation, money, a blood donor, identifying someone to accompany the woman and temporary family care in case of emergencies.^6^ Women should be knowledgeable about the key obstetric and newborn danger signs to reduce the occurrence of delay in seeking care. Such key danger signs in pregnancy, labour and delivery, post partum and in the newborn include severe vaginal bleeding, prolonged labour, convulsions, high fever, spasms and difficulty in breathing in the newborn among others.^6^ A systematic review of randomised controlled trials in developing countries showed that BP/CR strategies can indeed be effective.^7^

Mothers in Nigeria and other developing countries with high maternal and child mortality have been observed to have poor knowledge of obstetric and newborn danger signs, and BP/CR.^8^,^12^ Maternal educational status, place of delivery, access to mass media, antenatal care (ANC) attendance and being employed are some of the factors influencing their knowledge.^8 13^ Even pregnant women attending ANC in HFs had very poor knowledge of danger signs in pregnancy, labour and newborn.^14 15^ Community-based interventions which encourage prompt recognition and reaction to danger signs of complications during pregnancy and childbirth have the potential to prevent maternal mortality and birth complications.^16^ Employing participatory learning and action method among women’s groups has also been shown to be cost-effective in improving maternal and newborn health in low-resource settings.^17^

The study objectives were to determine the level of knowledge of BP/CR among women in Nigeria, provide multimethod educational interventions to improve their knowledge and attitude regarding the BP/CR strategy and assess the effect of the intervention thereafter. We hypothesised that there would be a statistically significant difference (preintervention and postintervention levels) in BP/CR knowledge between intervention (IG) and control groups (CG), and within the IG, postintervention.

## Methodology

### Study setting

The study was carried out in Ifako-Ijaiye (Intervention) and Kosofe (Control) local government areas (LGAs), Lagos State, Nigeria. They are 2 out of the 16 urban LGAs in Lagos State. These two LGAs were purposively chosen because the highest maternal deaths in Lagos State were recorded in these areas.^18^ They also share similar socioeconomic characteristics and have sufficient distance between them to prevent cross-interference of information thus reducing the possibility of contamination during the period of study. Ifako-Ijaiye has 14 political wards while Kosofe has 21. Each LGA has over 80 streets in total. Ifako Ijaiye LGA has a projected population (from the 2006 census and Lagos State growth rate of 3.2%) of 1 019 902 while Kosofe LGA has 1 280 646.^19^ Women and children form a good proportion of the population. At the time of the study, there were 11 primary healthcare centres (PHCs), 1 general hospital (GH) and about 118 private HFs in Ifako-Ijaiye while Kosofe had 12 PHCs, 1 GH and 127 private HFs. There are many registered and unregistered maternity homes and Traditional Birth Attendant (TBA) centres in both areas.

### Study population

The study population comprised mothers in the study area.

### Inclusion and exclusion criteria

Only women who delivered a baby in the past 2 years were interviewed. They were required to be residents in the study groups respectively for at least 1 year. Those unable to respond to questions appropriately and temporary visitors were excluded.

### Study design and sample size

This was a quasi-experimental study with Ifako-Ijaiye LGA as the intervention community and Kosofe LGA as the control community.

Sample size was determined using the formula for the comparison of proportions,^20^ and assumption of a preintervention and postintervention difference of 10.8% (P1–P2) in women’s knowledge of prolonged labour as a danger sign during delivery at baseline 11.4% and 22.2% postintervention,^21^ power at 90% (1.28), design effect 2. The minimum calculated sample size was further increased to make up for attrition, improperly filled questionnaires and to increase precision, and then rounded up to 650 for each group.

### Sampling methods

**Figure 1:**
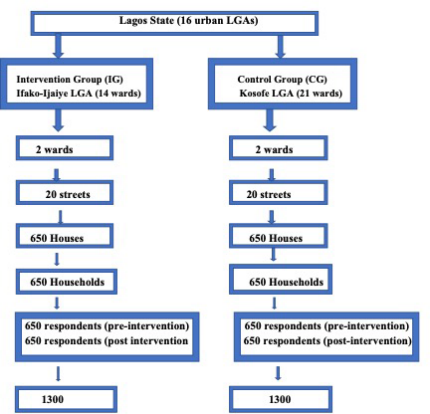
Stages of sampling for the selection of respondents. LGA, local government area.

Multistage sampling was used for selecting respondents. In the first stage, two wards were selected from the list of wards in each LGA using a simple random sampling technique. In the second stage, 10 streets in each ward were selected by simple random sampling. The streets served as clusters. For the third stage, research assistants (RAs) then moved from houses in a specified direction and identified women who met the inclusion criteria for the interview. This sampling method was applied in both the intervention and control communities (figure 1).

### Intervention

The intervention was carried out using a multimethod approach and lasted for 6 months (September 2019–March 2020). An adaptation of training and intervention strategies implemented in a previous study was used.^22^ Ten Maternal Health Volunteers (MHVs) were recruited from each study area and trained by the lead researchers for 3 weeks on the concept of BP/CR and facilitating participatory learning groups in the community. They were selected with the assistance of key stakeholders in the community based on specified criteria: (1) respected persons in the community, (2) permanent residents, (3) literate, (4) motivated to teach others about maternal health, (5) must not be an alternative maternity care provider (eg, TBA) and (6) willing and able to work on a volunteer basis. They were, however, given stipends to cover transportation and communication costs.

To kick off the women’s education, first, eligible women were mobilised to attend an awareness session at an accessible location within the community This was a one-time session where they were told about the proposed intervention and introduced to the concept of BP/CR. They were also informed of the selected MHVs who will work with them in smaller groups to improve their knowledge and attitude to BP/CR. To end the session, there was a drama presentation that communicated BP/CR and safe motherhood messages. Following this, initial mobilisation was the commencement of small group participatory learning facilitated by the trained MHVs who educated them on the danger signs in pregnancy, labour and delivery, postpartum, and in the newborn. They also covered the components of BP/CR, and the need to make birth and emergency plans well ahead of time. The group learning, divided into three learning modules held monthly for 3 months only. For ongoing learning, information, education and communication (IEC), materials in the form of handbills were distributed to women and posters containing information on key BP/CR messages were placed strategically on the walls of the participating women’s houses within the locality of the MHVs. An mHealth component was also included in the educational package. A short informative message on BP/CR was sent via short message service (SMS) and video via Whatsapp to all participating women who provided their phone numbers for the purpose. They were sent in the last month of the intervention to serve as reminders to re-enforce the message. We anticipated that the use of these platforms would be effective in this community as it is an urban community with high mobile/smartphone ownership. Interventions ended after the delivery of all components. The CG did not receive any of the educational interventions until after postintervention data collection.

During the preintervention (baseline) phase, baseline data were collected using questionnaires from eligible women in both communities. This was done over 2 months. Postintervention data collection was carried out 6 months after the educational intervention in the community was initiated. The same instrument used for the baseline questionnaire survey was used.

Community leaders and other stakeholders were consulted and their buy-in for the project was sought and obtained. This was necessary to increase community involvement in the project.

### Data collection methods and tools

Six RAs were trained and recruited for the administration of questionnaires. They were females with university degree qualifications, fluent in English, Yoruba and Pidgin English which were common languages spoken in the study areas and had good communication skills. They were trained on the objectives of the study, contents of the questionnaire and sampling procedures. They were also trained on obtaining informed consent and maintaining confidentiality.

Data were collected using structured, pretested, interviewer-administered questionnaires adapted from a validated tool from JHPIEGO.^23^ The questionnaire consisted of multiple sections which elicited information on their sociodemographic and obstetric characteristics. This was followed by questions that assessed their knowledge of obstetric and newborn danger signs, and basic components of BP/CR. Respondents gave spontaneous responses to all questions. They were then interviewed about the communication channels preferred for receiving BP/CR messages outside the HFs/health workers. The next section contained a set of statements with responses structured on a 3-point scale to assess their attitude to and perception of BP/CR. Respondents expressed their agreement, neutrality or disagreement with these statements. Information was also elicited on respondents’ personal experience with regard to the last pregnancy such as their utilisation of ANC, delivery and postnatal care (PNC) services and their exposure to BP/CR messages during ANC.

### Monitoring and evaluation and data quality assurance

There were monthly supervisory field visits to the RAs and MHVs during the period of study. Appropriate channels of communication were maintained with key community stakeholders throughout the project. Challenges were identified and resolved.

### Data management

Data were entered in Microsoft Excel and analysed with Epi Info V.3.5.1. and IBM-SPSS (Statistical Package for the Social Sciences) V.25 software (now known as Statistical Product and Service Solutions). Analyses were done between and within groups (four-way analysis): Between-group comparison of the IG and CG at baseline; within-group comparison of the IG before and after intervention; within-group comparison of the CG before and after intervention; and between-group comparison of the IG and CG postintervention.

Categorical data were summarised using proportions, while quantitative (numerical) data were summarised using mean and SD. Preintervention and postintervention samples were not paired in this study. χ² test was used to test for the differences in proportions between IG and CGs and between preintervention and postintervention phases, and also to test the associations between two categorical variables. Independent samples t-test was used to compare the knowledge and attitude scores between IG and CG and also between preintervention and postintervention phases. Mann-Whitney U test was used to compare the income between IG and CG. Multiple logistic regression was used to identify the predictors of good knowledge of BP/CR. The level of significance was set at p<0.05. Regression analysis was applied to the IG. Less than 0.1 was used as the cut-off p value for inclusion of variables into the logistic regression model after bivariate analysis.

### Measurements

Knowledge was scored and graded following adaptations from similar studies.^11 13^ Each spontaneous correct response mentioned earns the respondent one point. Only the key danger signs were used in the assessment of respondents’ knowledge of obstetric and newborn danger signs because they are common, easy to recognise and indicate a potentially severe problem.^23^

Pregnancy: Knowledge of at least two key danger signs in pregnancy—vaginal bleeding, severe headache, blurred vision, convulsions (four points).

Labour and delivery: Knowledge of at least two key danger signs during labour and delivery—severe vaginal bleeding, prolonged labour (lasting >12 hours), convulsion, retained placenta (not delivered 30 min after baby) (4 points).

Postpartum: Knowledge of at least two key danger signs—severe vaginal bleeding, foul-smelling vaginal discharge, high fever (3 points)

Newborn: Knowledge of at least two key danger signs—convulsions/spasms/rigidity, difficulty or fast breathing, very small baby, lethargy/unconsciousness (4 points).

All scores were summed up to obtain respondents’ knowledge of danger signs with scores ranging from 0 to 15 (100%). Those who scored less than 50% were classified as having inadequate knowledge while those who scored 50% or more were classified as having adequate knowledge.

For the knowledge of components of BP/CR, only the basic components were considered for assessment.^23^ Each spontaneous mention of the basic components attracted one point each namely: save money, identify HF/skilled provider, identify/arrange for transport and identify blood donor/donate blood. The minimum score was 0 and maximum 4 that is, 100%. Knowledge of at least three basic components of BP/CR was considered adequate knowledge of BP/CR components and less than 3, inadequate knowledge.

All scores from the danger signs and BP/CR subsections were then combined to assess overall knowledge of BP/CR,^11^ 0–19 (100%) graded as <50%—inadequate knowledge, 50% or more—adequate knowledge.

For the attitude, eight variables were used to assess respondent’s overall attitude towards BP/CR. ‘Agree’ responses to positive statements were scored 3, ‘not sure’ 2 and ‘disagree’ 1. Reverse scoring was applied to negative statements. The maximum score was 24 and minimum 8. A cut-off of 75% was used to grade respondents into having ‘positive’ attitude (≥75%) and ‘negative’ attitude (<75%).

## Results

A total sample size of 2600 was used for the study. These samples differed in certain personal characteristics. Table 1 shows that more respondents in the CG were single (p=0.046) while more respondents in the IG attained postsecondary educational level (p=0.001). The majority of the women in both communities had formal education and were employed.

**Table 1:** Sociodemographic and obstetric characteristics of respondents

Variable	Freq(%)	Freq(%)	χ^2^	P value
	Intervention	Control		
Age group (years)	n=650	n=650		
<20	7 (1.1)	16 (2.5)	16.803	0.001*
20–29	233 (35.8)	280 (43.1)		
30–39	373 (57.4	304 (46.8)		
≥40	37 (5.7)	50 (7.7)		
Mean age	31.2±5.4	30.4±6.0	T=2.700	0.007*
Marital status				
Married/cohabiting	580 (89.2)	570 (87.7)	6.153	0.046*
Never married	55 (8.5)	76 (11.7)		
Widowed/separated/divorced	15 (2.3)	4 (0.6)		
Ethnicity				
Yoruba	473 (72.8)	397 (61.1)	27.896	<0.001*
Igbo	106 (16.3)	119 (18.3)		
Hausa	8 (1.2)	9 (1.4)		
Others†	63 (9.7)	125 (19.2)		
Religion				
Christianity	451 (69.4)	452 (69.2)	0.004	0.952
Islam	199 (30.6)	198 (30.5)		
Education				
No formal education	16 (2.5)	11 (1.7)	16.610	0.001*
Primary	84 (12.9)	120 (18.5)		
Secondary	391 (60.2)	409 (62.9)		
Postsecondary	159 (24.5)	110 (16.9)		
Employment status				
Employed	444 (68.3)	538 (82.8)	36.784	<0.001*
Unemployed	206 (31.7)	112 (17.2)		
Monthly income (₦)				
<₦30 000	308 (47.4)	233 (35.8)	71.026	<0.001*
₦30 000–₦39 000	59 (9.1)	154 (23.7)		
₦40 000–₦49 000	35 (5.4)	86 (13.2)		
≥₦50 000	96 (14.8)	138 (21.3)		
NR	*152* (*23.4*)	*39* (*6.0*)		
Mean (SD) income	33 175 (46,684)	35 810 (24,400)	MWU=11 190;	<0.001*
Median income	20,0000	30 000		
Parity				
1	113 (17.4)	154 (23.7)	19.511	0.001*
2	155 (23.8)	199 (30.6)		
3	111 (17.1)	151 (23.2)		
4	53 (8.2)	88 (13.5)		
≥5	11 (1.7)	57 (88)		
NR	*207* (*31.8*)	*1* (*0.2*)		
			MWU=1314	0.013*
No of living children				
1	161 (24.8)	139 (21.4)	16.822	0.002*
2	177 (27.2)	175 (26.9)		
3	131 (20.2)	117 (18.0)		
4	50 (7.7)	71 (10.9)		
≥5	13 (2.0)	36 (5.5)		
NR	*118* (*18.2*)	*112* (*17.2*)		
			MWU=1302;	0.008*

* Statistically significant.

† Fishers exact p T Students t test OOthers: Ijaw, Ikwere, Kanuri, Isoko, etc.

MWU, Mann-Whitney UNR, no response

Table 2 shows that most of the respondents in both groups had their last ANC in HFs, especially private ones (p=0.240). Counselling on the basic components of BP/CR was also very poor. In their last pregnancy, most of the respondents had facility delivery and had PNC check-up by health worker within hours of birth.

**Table 2:** Utilisation of maternity care

Variable	Frequency (%)		χ^2^	P value
	Intervention	Control		
	n=650	n=650		
Place of last ANC				
Did not have ANC	16 (2.8)	10 (1.5)	14.018	0.003*
Only health facility	514 (79.1)	492 (75.7)		
Only TBA	27 (4.2)	55 (8.5)		
Both HF and TBA	33 (5.1)	48 (7.4)		
No response	60 (9.2)	45 (6.9)		
Hospital of last ANC	(n=514)	(n=492)		
Private	305 (46.9)	298 (45.8)	2.857	0.240
Public	165 (25.4)	193 (29.7)		†0.182
Public and private	1 (0.2)	0 (0)		
NR	70 (11.7)	49 (7.5)		
Respondent advised on:				
Danger signs				
Yes	442 (68.0)	511 (78.6)	18.716	<0.001*
No	208 (32.0)	139 (21.4)		
Where to go if observes danger sign				
Yes	414 (63.7)	473 (72.8)	12.353	<0.001*
No	236 (36.3)	177 (27.2)		
Where to give birth				
Yes	439 (67.5)	358 (55.1)	21.276	<0.001*
No	211 (32.5)	292 (44.9)		
Transport arrangement				
Yes	167 (25.7)	77 (11.8)	40.867	<0.001*
No	483 (74.3)	573 (88.2)		
Fund/finance arrangement				
Yes	281 (43.2)	246 (37.8)	3.909	0.048*
No	369 (56.8)	404 (62.2)		
Blood donor arrangement				
Yes	152 (23.4)	76 (11.7)	30.721	<0.001*
No	498 (76.6)	574 (88.3)		
Health provider to deliver baby				
Yes	275 (42.3)	9 (1.4)	344.596	<0.001*
No	375 (57.7)	641 (98.6)		
Place of last delivery				
Health facility	514 (79.1)	464 (71.4)	26.301	<0.001*
TBA	56 (8.6)	86 (13.2)		
Religious home	40 (6.2)	58 (8.9)		
Respondent’s home	13 (2.0)	31 (4.8)		
Others‡	27 (4.2)	11 (1.7)		
Had PNC within 6 wks				
Yes	557 (85.7)	618 (95.1)	32.935	<0.001*
No	93 (14.3)	32 (4.9)		
Time of first PNC check	n=557	n=618		
Hours	530 (95.2)	586 (94.8)	5.512	0.064
Days	12 (2.2)	28 (4.5)		†0.051
Weeks	1 (0.2)	3 (0.5)		
NR	14 (2.5)	1 (0.2)		
PNC check carried out by	n=557	n=618		
Health worker	491 (88.2)	537 (86.9)	12.121	0.002*
TBA	27 (4.8)	61 (9.9)		
Others§	28 (5.0)	20 (3.2)		
NR	11 (2.0)	0 (0)		

* Statistically significant.

† Fisher’s exact.

‡ Others: Ccar, Mmarket.

§ Others: Mmother, Nneighbour, Aaunty, Ssister, Mmother- in- law.

ANCantenatal careHFhealth facilityMWU, Mann-Whitney UNR, no responsePNCpostnatal careTBAtraditional birth attendant

Details of the respondents’ preintervention and postintervention knowledge of danger signs and BP/CR components are shown in table 3. The least known key danger signs (IG vs CG) were during pregnancy preintervention was convulsion 12.6% vs 4.0%, postintervention 30.3% vs 5.1% (p<0.001); during delivery preintervention was also convulsion 17.1% vs 4.9% postintervention 33.1% vs 5.1% (p<0.001); for the post partum period preintervention, it was foul swelling vaginal discharge 11.1% vs 7.1%, postintervention 30.2% vs 12.2% (p<0.001); and for the newborn preintervention, lethargy/unconsciousness 8.3% vs 0.6%, postintervention 20.3% vs 1.9% (p<0.001). Table 4 shows that preintervention, both groups had overall inadequate knowledge of danger signs. A higher proportion had adequate knowledge of danger signs during labour and delivery (IG vs CG) 47.1% vs 34.6% preintervention and 64.5% vs 36.6% postintervention. Regarding knowledge of basic components of BP/CR, IG recorded a significant increase in adequate knowledge from 17.2% preintervention to 54% postintervention in the IG (p<0.001) but no significant change was observed in the inadequate knowledge of the CG. For overall knowledge of BP/CR, only 9.4% of IG, and 0.2% of CG had adequate knowledge. But postintervention, there was a significant improvement to 52% in the IG representing an increment of 42.6% (p<0.001) but not for CG where respondents’ poor knowledge showed no significant positive change with an increment of only 0.3% to a final 0.5% (Fisher’s exact p=0.624).

**Table 3:** Knowledge of danger signs and components of BP/CR—comparison between and within groups

	Between-groups preintervention				Between-groups postintervention				Within-groupχ^2^; p value			
	Frequency (%)		χ^2^	P value	Frequency (%)		χ^2^	P value				
	IG	CG			IG	CG			χ^2^	P value	χ^2^	P value
	n=650	n=650			n=650	n=650			IG		CG	
Danger signs in pregnancy												
Bleeding	396 (60.9)	286 (44)	37.321	<0.001*	515 (79.2)	436 (67.1)	24.445	<0.001*	51.948	<0.001*	70.091	<0.001*
Severe headache	93 (14.3)	79 (12.2)	1.313	0.252	355 (54.6)	67 (10.3)	291.019	<0.001*	233.791	<0.001*	1.111	0.292
Blurred vision	97 (14.9)	79 (12.2)	2.129	0.145	224 (34.5)	22 (3.4)	204.019	<0.001*	66.721	<0.001*	34.878	<0.001*
Convulsions	82 (12.6)	26 (4.0)	31.644	<0.001*	197 (30.3)	33 (5.1)	142.076	<0.001*	60.354	<0.001*	0.870	0.351
Swollen hands/face	139 (21.4)	131 (20.2)	0.299	0.584	313 (48.2)	76 (11.7)	206.050	<0.001*	102.685	<0.001*	17.381	<0.001*
High fever	167 (25.7)	216 (33.2)	8.887	0.003*	370 (56.9)	164 (25.2)	134.868	<0.001*	130.748	<0.001*	10.005	0.002*
Loss of consciousness	75 (11.5)	98 (15.1)	3.527	0.060	219 (33.7)	125 (19.2)	34.929	<0.001*	91.143	<0.001*	3.946	0.047
Difficulty in breathing	73 (11.2)	61 (9.4)	1.198	0.274	244 (37.5)	28 (4.3)	216.915	<0.001*	121.990	<0.001*	13.135	<0.001*
Severe weakness	193 (29.7)	208 (32.0)	0.811	0.368	393 (60.5)	180 (27.7)	141.584	<0.001*	124.282	<0.001*	2.880	0.090
Severe abdominal pain	158 (24.3)	148 (22.8)	0.427	0.513	393 (60.5)	145 (22.3)	195.034	<0.001*	173.959	<0.001*	0.040	0.842
Accelerated/reduced fetal movement	71 (10.9)	14 (2.2)	40.898	<0.001*	173 (26.6)	23 (3.5)	135.176	<0.001*	52.492	<0.001*	2.253	0.133
Water breaks without labour	115 (17.7)	25 (3.8)	64.840	<0.001*	256 (39.4)	68 (10.5)	145.300	<0.001*	74.988	<0.001*	21.414	<0.001*
Others	58 (8.9)	99 (15.2)	12.173	<0.001*	64 (9.8)	28 (4.3)	15.160	<0.001*	0.326	0.568	43.990	<0.001*
None	11 (1.7)	1 (0.2)	8.411	0.004*	0 (0)	0 (0)	–	–	11.094	0.001*	1.001	0.317
Don’t know	38 (5.8)	42 (6.5)	0.213	0.644	7 (1.1)	21 (3.2)	7.154	0.007*	22.121	<0.001*	7.357	0.007*
Danger signs during delivery												
Severe bleeding	322 (49.5)	318 (48.9)	0.049	0.824	495 (76.2)	280 (43.1)	147.693	<0.001*	98.598	<0.001*	4.468	0.035*
Prolonged labour	405 (62.3)	307 (47.2)	29.822	<0.001*	389 (59.8)	394 (60.6)	0.080	0.777	0.363	<0.001*	23.433	<0.001*
Convulsion	111 (17.1)	32 (4.9)	49.037	<0.001*	215 (33.1)	33 (5.1)	165.051	<0.001*	44.283	<0.001*	0.016	0.899
Retained placenta	206 (31.7)	170 (26.2)	4.849	0.028*	285 (43.8)	153 (23.5)	59.994	<0.001*	20.425	<0.001*	1.191	0.275
Severe headache	148 (22.8)	51 (7.8)	55.827	<0.001*	360 (55.4)	30 (4.6)	398.901	<0.001*	145.220	<0.001*	5.806	0.016
High fever	112 (17.2)	67 (10.3)	13.119	<0.001*	365 (56.2)	98 (15.1)	239.144	<0.001*	211.966	<0.001*	6.671	0.010
Loss of consciousness	120 (18.5)	116 (11.2)	0.083	0.773	276 (42.5)	102 (15.7)	122.933	<0.001*	88.375	<0.001*	1.080	0.299
Others	32 (4.9)	73 (11.2)	17.416	<0.001*	49 (7.5)	21 (3.2)	11.837	0.001*	3.805	0.051	31.008	<0.001*
None	16 (2.5)	0 (0)	16.199	<0.001*	1 (0.2)	0 (0)	1.001	0.317	13.411	<0.001*	–	–
Don’t know	48 (7.4)	39 (6.0)	0.998	0.318	13 (2.0)	35 (5.4)	10.470	0.001*	21.071	<0.001*	0.229	0.632
Danger signs during postpartum period												
Severe bleeding	385 (59.2)	467 (71.8)	22.901	<0.001*	557 (85.7)	540 (83.1)	1.687	0.194	114.042	<0.001*	23.480	<0.001*
Foul swelling vaginal discharge	72 (11.1)	46 (7.1)	6.301	0.012*	196 (30.2)	79 (12.2)	63.133	<0.001*	72.272	<0.001*	9.639	0.002*
High fever	103 (15.8)	60 (9.2)	12.970	<0.001*	303 (46.6)	93 (14.3)	160.147	<0.001*	143.265	<0.001*	8.067	0.005*
Severe headache	210 (32.3)	82 (12.6)	72.364	<0.001*	347 (53.4)	22 (3.4)	399.700	<0.001*	58.958	<0.001*	37.625	<0.001*
Blurred vision	138 (21.2)	52 (8.0)	45.589	<0.001*	267 (41.1)	10 (1.5)	303.008	<0.001*	59.682	<0.001*	29.876	<0.001*
Convulsions	94 (14.5)	44 (6.8)	20.267	<0.001*	198 (30.5)	38 (5.8)	132.535	<0.001*	47.771	<0.001*	0.469	0.494
Swollen hands/face	92 (14.2)	19 (2.9)	52.491	<0.001*	287 (44.2)	46 (7.1)	234.480	<0.001*	141.616	<0.001*	11.806	0.001*
Loss of consciousness	48 (7.4)	124 (19.1)	38.702	<0.001*	216 (33.3)	71 (10.9)	94.013	<0.001*	134.152	<0.001*	16.947	<0.001*
Difficulty in breathing	86 (13.2)	7 (1.1)	72.278	<0.001*	277 (42.6)	10 (1.5)	318.768	<0.001*	139.432	<0.001*	0.536	0.464
Severe weakness	171 (26.3)	262 (40.3)	28.676	<0.001*	449 (69.1)	219 (33.7)	162.894	<0.001*	238.305	<0.001*	6.102	0.014*
Others	42 (6.5)	65 (10.0)	5.387	0.020*	42 (6.5)	15 (2.3)	13.376	<0.001*	0.000	1.000	33.299	<0.001*
None	14 (2.2)	1 (0.2)	11.398	0.001*	2 (0.3)	0 (0)	2.003	0.157	9.112	0.003*	1.001	0.317
Don’t know	110 (16.9)	44 (6.8)	32.087	<0.001*	10 (1.5)	20 (3.1)	3.412	0.065	91.808	<0.001*	9.466	0.002*
Serious health problems that can occur during the first 7 days of birth												
Convulsions/spasms/rigidity	156 (24.0)	86 (13.2)	24.879	<0.001*	183 (28.2)	27 (4.2)	138.212	<0.001*	2.909	0.088	33.738	<0.001*
Difficult or fast breathing	299 (46.0)	170 (26.2)	55.507	<0.001*	458 (70.5)	102 (15.7)	397.579	<0.001*	79.954	<0.001*	21.498	<0.001*
Very small baby	113 (17.4)	33 (5.1)	49.382	<0.001*	369 (56.8)	21 (3.2)	443.604	<0.001*	216.084	<0.001*	2.882	0.095
Lethargy/unconsciousness	54 (8.3)	4 (0.6)	45.116	<0.001*	132 (20.3)	6 (l.9)	128.706	<0.001*	38.171	<0.001*	0.403	0.525
Yellow skin/eye colour	410 (63.1)	403 (77.4)	31.822	<0.001*	532 (81.8)	584 (89.8)	17.119	<0.001*	57.839	<0.001*	36.839	<0.001*
Poor sucking or feeding	154 (23.7)	72 (11.1)	36.013	<0.001*	317 (48.8)	36 (5.5)	307.066	<0.001*	88.459	<0.001*	13.087	<0.001*
Pus, bleeding from cord	95 (14.6)	200 (30.8)	48.343	<0.001*	264 (40.6)	165 (25.4)	34.099	<0.001*	109.909	<0.001*	4.666	0.031*
Skin lesions or blisters	51 (7.8)	55 (8.5)	0.164	0.685	247 (38.0)	33 (5.1)	208.455	<0.001*	167.252	<0.001*	5.899	0.015*
Red or swollen eyes with pus	32 (4.9)	23 (3.5)	1.538	0.215	174 (26.8)	10 (1.5)	170.274	<0.001*	116.315	<0.001*	5.255	0.022*
Others	30 (4.6)	65 (10.0)	13.911	<0.001*	34 (5.2)	30 (4.6)	0.263	0.608	0.263	0.608	13.911	<0.001*
None	16 (2.5)	6 (0.9)	4.624	0.032*	0 (0)	0 (0)	–	–	16.199	0.210	6.028	0.014*
Don’t know	25 (3.8)	22 (3.4)	0.199	0.656	17 (2.6)	6 (0.9)	5.356	0.021*	1.575	<0.001*	9.344	0.002*
Basic components of BP/CR												
Identify mode of transport	83 (12.8)	13 (2.0)	55.111	<0.001*	348 (53.5)	15 (2.3)	423.824	<0.001*	243.746	<0.001*	0.146	0.702
Save money	338 (52.0)	477 (73.4)	63.544	<0.001*	459 (70.6)	400 (61.5)	11.946	0.001*	47.477	<0.001*	20.777	<0.001*
Identify blood donor	83 (12.8)	13 (2.0)	55.111	<0.001*	177 (27.2)	27 (4.2)	130.823	<0.001*	42.481	<0.001*	5.056	0.025*
Identify skilled provider	397 (61.1)	194 (29.8)	127.850	<0.001*	454 (69.8)	20 (40.9)	196.962	<0.001*	11.054	0.001*	0.178	0.673
Identify someone to accompany her for delivery	54 (8.3)	1 (0.2)	53.329	<0.001*	91 (14.0)	7 (1.1)	77.870	<0.001*	10.627	0.001*	4.528	0.033*
Temporary family care	53 (8.2)	1 (0.2)	52.244	<0.001*	287 (44.2)	5 (0.8)	351.235	<0.001*	218.085	<0.001*	2.679	0.102
Others	64 (9.8)	5 (0.8)	53.277	<0.001*	164 (25.2)	76 (11.7)	39.572	<0.001*	53.188	<0.001*	66.370	<0.001*

* Statistically significant.

BPbirth preparednessCG, control groupCRcomplications readinessIG, intervention group

**Table 4:** Overall knowledge of danger signs and basic components of BP/CR- comparison between and within groups

Knowledge	Between-groups preintervention				Between-groups postintervention				Within-groupχ^2^; p value	
	Frequency (%)		χ^2^	P value	Frequency (%)		χ^2^	P value		
	IG	CG			IG	CG				
	n=650	n=650			n=650	n=650			IG	CG
DS in pregnancy										
Adequate	116 (17.8)	56 (8.6)	24.122	<0.001*	376 (57.8)	37 (5.7)	407.821	<0.001*	221.06; <0.001*	4.181; 0.041*
Inadequate	534 (82.2)	594 (91.4)			274 (42.2)	613 (94.3)				
DS during labour and delivery										
Adequate	306 (47.1)	224 (34.6)	21.419	<0.001*	419 (64.5)	238 (36.6)	100.815	<0.001*	39.819; <0.001*	0.658; 0.417
Inadequate	344 (52.9)	426 (65.5)			231 (35.5)	412 (63.4)				
DS post partum										
Adequate	92 (14.2)	81 (12.5)	0.807	0.369	327 (50.3)	128 (19.7)	133.900	<0.001*	194.486; <0.001*	12.594; <0.001*
Inadequate	558 (85.8)	569 (87.5)			323 (49.7)	522 (80.3)				
DS in newborn										
Adequate	167 (25.7)	32 (4.9)	108.136	<0.001*	386 (59.4)	17 (2.6)	489.664	<0.001*	150.934; <0.001*	4.772; 0.029*
Inadequate	483 (74.3)	618 (95.1)			264 (40.6)	633 (97.4)				
BP/CR basic components										
Adequate	112 (17.2)	22 (3.4)	67.395	<0.001*	351 (54.0)	29 (4.5)	385.553	<0.001*	191.616; <0.001*	1.000; 0.317
Inadequate	538 (82.8)	628 (96.6)			299 (46.0)	621 (95.5)				
Overall knowledge score of BP/CR (%)										
Adequate	61 (9.4)	1 (0.2)			338 (52.0)	3 (0.5)			277.463; <0.001*	1.003; 0.3170.624†
Inadequate	589 (90.6)	649 (99.8)	60.972	<0.001*	312 (48.0)	647 (99.5)	446.129	<0.001*		
Mean knowledge score	25.4±17.4	20.2±9.6	T=6.758; <0.001*		43.4±23.1	20.7±9.0	T=23.364; <0.001*		T=15.828; <0.001*	T=0.987; 0.324

* Statistically significant Aadequate knowledge ≥50%; Iinadequate knowledge <50%.

† Fisher’s exact.

BPbirth preparednessCGcontrol groupCRcomplications readinessDSdanger signs IGintervention group

Most respondents in both IG and CG had positive attitude to BPCR and the intervention had no significant influence on this in the IG (p=0.504) (table 5). Asides HFs/workers, respondents in both groups received information on BP/CR mainly from mass media (television and radio). These were also their preferred sources. Postintervention, preference for IEC materials such as fliers and for internet/social media as sources of information increased significantly in the IG and decreased significantly in the CG. For fliers (pre vs post), IG 1.7% vs 32.8%, (p<0.001), CG 6.6% vs 3.8% (p=0.025). For internet/social media (pre vs post), IG 12.3% vs 32.8% (p<0.001), CG 24.3% vs 13.8% (p<0.001) (not shown in the tables).

**Table 5:** Attitude to BP/CR—comparison between and within groups

Attitude	Between-groups preintervention				Between-groups postintervention				Within-groupχ^2^; p value	
	Frequency (%)		χ^2^	P value	Frequency (%)		χ^2^	P value		
	IG	CG			IG	CG				
	n=650	n=650			n=650	n=650			IG	CG
A woman should plan ahead where to give birth										
Agree	643 (98.9)	637 (98.0)	5.095	0.078	647 (99.5)	642 (98.9)	2.886	0.236	6.512; 0.039*0.039†	1.212; 0.546*0.545
Not sure	7 (1.1)	8 (1.2)		*0.082	1 (0.2)	5 (0.8)		*0.208		
Disagree	0 (0)	5 (0.8)			2 (0.3)	3 (0.5)				
A woman should plan ahead how she will get to place of delivery										
Agree	622 (95.7)	567 (87.2)	30.445	<0.001†	629 (96.8)	606 (93.2)	11.745	0.003†	3.306; 0.191	13.677; 0.001†
Not sure	17 (2.6)	42 (6.5)			17 (2.6)	25 (3.9)				
Disagree	11 (1.7)	41 (6.3)			4 (0.6)	19 (2.9)				
It is not necessary for husband/spouse to accompany woman to ANC										
Agree	327 (50.3)	369 (56.8)	12.097	0.002†	262 (40.3)	529 (81.4)	283.699	<0.001†	160.032; <0.001†	94.667; <0.001†
Not sure	59 (9.1)	76 (11.7)			247 (38.0)	23 (3.5)				
Disagree	264 (40.6)	205 (31.5)			141 (21.7)	98 (15.1)				
When women do not deliver in HF, it is mainly because it is too expensive										
Agree	213 (32.8)	312 (48.0)	39.967	<0.001†	102 (15.7)	270 (41.5)	130.542	<0.001†	60.254; <0.001†	6.272; 0.043†
Not sure	114 (17.5)	58 (8.9)			191 (29.4)	75 (11.5)				
Disagree	323 (49.7)	280 (43.1)			357 (54.9)	305 (46.9)				
When women do not deliver in HF, it is mainly because it is too difficult to reach there										
Agree	162 (24.9)	115 (17.7)	31.975	<0.001†	67 (10.3)	159 (24.5)	58.568	<0.001†	54.735; <0.001†	22.556; <0.001†
Not sure	110 (16.9)	62 (9.5)			173 (26.6)	98 (15.1)				
Disagree	378 (58.2)	473 (72.8)			410 (63.1)	393 (60.5)				
When women do not deliver in HF, it is mainly due to staff disrespect										
Agree	228 (35.1)	203 (31.2)	6.463	0.040†	191 (29.4)	148 (22.8)	52.730	<0.001†	16.124; <0.001†	13.180; 0.001†
Not sure	82 (12.6)	64 (9.8)			134 (20.6)	59 (9.1)				
Disagree	340 (52.3)	383 (58.9)			325 (50.0)	443 (68.2)				
It is not necessary for husband/spouse to accompany woman for delivery										
Agree	302 (46.5)	268 (41.2)	3.640	0.162	401 (61.7)	344 (52.9)	10.378	0.006†	30.380; <0.001†	18.030; <0.001†
Not sure	22 (3.4)	23 (3.5)			15 (2.3)	16 (2.5)				
Disagree	326 (50.2)	359 (55.2)			234 (36.0)	290 (44.6)				
Giving birth is not only woman’s matter, husbands/partners have a lot to contribute										
Agree	623 (95.8)	616 (94.8)	8.828	0.012†	598 (92.0)	630 (96.9)	16.914	<0.001†	11.441; 0.003†	11.080; 0.004†
Not sure	14 (2.2)	29 (4.5)			16 (2.5)	10 (1.5)				
Disagree	13 (2.0)	5 (0.8)			36 (5.5)	10 (1.5)				
Overall attitude score (%)										
Positive attitude	348 (53.5)	359 (55.2)	0.375	0.540	360 (55.4)	352 (54.2)	0.199	0.656	0.447; 0.504	0.152; 0.697
Negative attitude	302 (46.5)	291 (44.8)			290 (44.6)	298 (45.8)				
Mean score	71.8±17.3	71.2±16.9	T=0.619; 0.536		71.9±11.9	68.5±16.9	T=4.452; <0.001†		T=0.15; 0.879	T=2.990; 0.003†

* Fisher’s exact Ppositive attitude ≥75 and Nnegative attitude <75.

† Statistically significant.

ANCantenatal careBPbirth preparednessCGcontrol groupCRcomplications readinessHFhealth facilityIGintervention group

Bivariate analysis showed some variables as significant factors associated with knowledge viz: age (p=0.004), ethnicity (p=0.001), employment status (p=0.016), educational level (p=0.014), monthly income (Fisher’s exact p<0.001), number of children (Fisher’s exact p<0.001) and time of last delivery (p=0.008) (online supplemental material 1).

Table 6 shows the predictors of adequate knowledge of BPCR viz: being of Yoruba tribe (adjusted OR (AOR) 2.83; 95% CI 1.06 to 7.54), being employed (AOR 1.31; 95% CI 1.04 to 5.87) and having a baby in the 6 months prior to the study (AOR 2.62; 95% CI 1.31 to 5.24). The odds of having adequate knowledge of BPCR were about three times higher among Yoruba women than those from other tribes. Women who were employed had almost three times higher odds of having adequate knowledge and those who were nursing mothers were almost three times more likely to be knowledgeable about danger signs and components of BPCR than those who had their babies over 6 months prior to the study.

**Table 6:** Multiple logistic regression of adequate knowledge of BPCR on associated factors

Predictor variable	β	SE	Wald	P value	AOR	95% CI	
						Lower	Upper
Constant	−6.021	1.207	24.905	<0.001*			
Age (years)							
<30	0.536	0.376	2.030	0.154	1.71	0.82	3.57
≥30					1		
Ethnicity							
Yoruba	1.040	0.501	4.314	0.038*	2.83	1.06	7.54
Others					1		
Educational level							
>Primary	1.659	1.031	2.587	0.108	5.25	0.70	39.66
≤Primary					1		
Employment status							
Employed	0.903	0.442	4.179	0.041*	2.47	1.04	5.87
Unemployed					1		
No of living children							
≥3	−0.524	0.447	1.375	0.241	0.59	0.25	1.42
<3					1		
Last delivery (months)							
≤6	0.964	0.353	7.444	0.006*	2.62	1.31	5.24
>6					1		

* Statistically significant.

AORadjusted ORBPbirth preparednessCRcomplications readiness

## Discussion

This study used a quasi experimental design involving a multimethod educational package at the community level to improve women’s knowledge and attitude to BP and CR. The respondents in both intervention and control arms had inadequate knowledge of danger signs and basic components of BP/CR at baseline. This inadequate knowledge, however, improved significantly in the IG postintervention. Their attitude to BP/CR was not affected by the intervention, though both groups mostly had positive attitude initially.

Similar studies reported that respondents had poor knowledge of obstetric and newborn danger signs at baseline such as those in Ghana,^24^ northern Nigeria,^25^ Tanzania,^26^,^28^ India,^29^ Eritrea^22^ and Nepal.^30^ In a study in Uganda, respondents’ baseline knowledge of danger signs in pregnancy (study and CGs) and post partum (study group) was high but low for newborn danger signs in both groups.^31^ Also in another rural study location in Bangladesh, women’s knowledge of newborn danger signs was high but low for the obstetric period at baseline.^32^ Our respondents had better knowledge of danger signs during labour and delivery. These aforementioned studies also recorded improvements in knowledge of danger signs after the introduction of some form of intervention.

The improved capacity for the women increases the chances of recognising these danger signs prompting the seeking of appropriate care thereby reducing delays associated with maternal and child deaths. The inadequate knowledge levels preintervention also point to deficits from the healthcare providers since most of them used formal healthcare in the previous pregnancy. Health worker training interventions to improve communication during ANC using job aids and guidelines were instrumental in improving women’s knowledge of BP/CR as reported by researchers in west and east Africa.^33 34^

The marked improvement in knowledge of obstetric and newborn danger signs and components of BP/CR seen among the respondents could be attributed to the multiple educational methods applied in the intervention. Being an urban setting, we leveraged high mobile/smartphone ownership to impart knowledge. Other similar studies were mainly rural, where interpersonal counselling was the mainstay, and improvements were also recorded.^2224 27^,^32 35^ The use of SMS messages was also applied in another urban setting in Tanzania where a very significant improvement in knowledge of BP/CR was recorded in the IG.^26^ Participatory community-based approach has been promoted by WHO and some studies have reported varying degrees of success.^4 22 30 36 37^

The most common obstetric danger signs known by our respondents were bleeding (pregnancy), severe bleeding and prolonged labour while they hardly knew foul-smelling vaginal discharge. Some also mentioned convulsions and fast breathing for the newborn. Their inadequate knowledge of basic components of BP/CR was also evident, except for saving money and identifying skilled provider which both had more mentions. The majority of them used formal maternity care services and yet were still deficient in knowledge of BP/CR. There appears to be gaps in the quality of services received at the HFs. Poor quality reproductive, maternal, newborn and child healthcare services and weak health systems contribute to the slow pace of progress and threaten the achievement of SDGs by 2030.^38^

A similar pattern of knowledge of danger signs was reported in the Ghanian study.^24^ In that study, only 51.1% of respondents could mention at least three of the danger signs and symptoms during pregnancy. In Tanzania, a large proportion of women in both IG and CGs also could not recall obstetric and newborn danger signs.^28^ Very poor knowledge of BP/CR components was reported in another study in Uganda and it did not improve significantly after intervention. Respondents in that study also commonly mentioned saving money as a basic component of BPCR.^31^

Some sociodemographic, economic and obstetric factors namely age, ethnicity, employment status, educational level, monthly income/allowance, number of children and time of last delivery were significant in bivariate analysis. On further analysis, four predictors of adequate knowledge of BP/CR were revealed—ethnicity, employment status and time of last delivery. These results are similar to previous studies, though with some differences. In Tanzania, being of the Mambwe ethnic group predicted improved knowledge of BP/CR among pregnant women.^28^ The odds of having adequate knowledge were three times higher among our respondents of Yoruba tribe extraction. Lagos state is mainly inhabited by Yorubas, and this may be the common language of delivering health messages generally, including HFs. Thus, non-Yorubas may be missing some important BP/CR information. In northern Nigeria, women who were employed had significantly better knowledge of BP/CR.^8^ Similarly, the odds of having adequate knowledge were about 2.5 times higher among our respondents who were employed. By virtue of their employment, some women may be more exposed to BP/CR content, hence their better knowledge. They are also more financially empowered to access formal maternity care services and all the benefits including BP/CR counselling. Those who delivered within 6 months prior to the study probably still had fresh memory of the messages they received during their maternity care contacts with health workers. Having adequate ANC and PNC services, in addition to other factors like older maternal age predicted good knowledge of danger signs in Ghana.^24^ Higher educational level, younger age and living with a partner predicted good knowledge of danger signs in Uganda.^31^ In east Africa, women with higher educational level had significantly improved knowledge of BP/CR.^26^ We assume that women were not receiving sufficient BP/CR messages even from the HFs, else, they would not have had such inadequate knowledge. The approach we adopted for the intervention was community-oriented, facilitating knowledge transfer of key messages outside the formal settings, where many women may not go. In addition to the women, significant others such as men and older women who are involved in decision-making on maternity care issues were also exposed to the same messages. They could join the participatory groups led by the MHVs, or also learn from the educative posters, handbills, SMS and video clips on BPCR circulated on WhatsApp platform. Having adequate knowledge of BP/CR should result in better utilisation of formal maternity health services which will go a long way in achieving results with maternal and newborn deaths reduction in Nigeria and other LMICs.

In all, beyond the effectiveness of the intervention, we need to look at sustainable mechanisms for improving knowledge of BP/CR. Quality of safe motherhood messages delivered at HFs, and training and retainment of MHVs in the community are some areas to be considered.

### Strengths and weaknesses

Evidence from this research was strengthened by a combination of multimethod educational intervention implemented at the community level, use of a CG, the prospective nature of the study, scientifically sound sampling methodology, measurement of knowledge of ‘key’ danger signs and components of BPCR and spontaneous responses to questions on knowledge of BP/CR. Non-pairing of participants, non-blinding and possibility of recall bias were limitations.

## Conclusion

This combination of participatory learning sessions, IEC materials and mHealth significantly improved women’s very poor knowledge of BP/CR in the IG. Similar programmes can be replicated in a sustainable manner to improve knowledge of BP/CR and empower women to make life-saving decisions. Further research can examine the roles of health system quality of care and economic exclusion in the inadequate knowledge of BP/CR among women in Nigeria.

## supplementary material

10.1136/bmjph-2023-000203 online supplemental material 1

## Data Availability

All data relevant to the study are included in the article or uploaded as online supplemental information.
