# Supplementary material for: Effectiveness of multimethod, community-based educational interventions on the knowledge and attitude to birth preparedness and complications readiness among women in southwest Nigeria
Source: BMJ Public Health. 2024 Jun 10;2(1):e000203. doi: 10.1136/bmjph-2023-000203 (PMC11812750; doi:10.1136/bmjph-2023-000203)
Supplement: online supplemental material 1 [file bmjph-2-1-s001.pdf]

Associations between sociodemographic and obstetric characteristics and knowledge

Variable	Knowledge (%)			χ^2	p-value
	Adequate	Inadequate	Total		
	n = 61	n = 589	n = 650		
**Age (year)**					
<30	33 (13.8)	207 (86.3)	240	8.526	0.004*
⩾30	28 (6.8)	382 (93.2)	410		
**Marital status**					
Married	56 (9.7)	524 (90.3)	580	0.464	0.496
Not married	5 (7.1)	65 (92.9)	70		
**Ethnicity**					
Yoruba	55 (11.6)	418 (88.4)	473	10.279	0.001*
Others	6 (3.4)	171 (96.6)	177		
**Religion**					
Christianity	40 (89)	411 (91.1)	451	0.460	0.498
Islam	21 (10.6)	178 (89.4)	199		
**Employment status**					
Employed	50 (11.3)	394 (88.7)	444	5.802	0.016*
Unemployed	11 (5.3)	195 (94.7)	206		
**Educational level**					
No formal education	0 (0)	15 (100)	15	10.631	0.014*
Primary	3 (3.6)	81 (96.4)	84		
Secondary	48 (12.3)	343 (87.7)	391		
Post-secondary	10 (6.3)	149 (93.7)	159		
**Monthly income (₦)**					
<30,000	31 (10.1)	277 (89.9)	308	15.759	†<0.001*
30,000-39,000	0 (0)	59 (100)	59		
40,000-49,000	0 (0)	35 (100)	35		
⩾50,000	2 (2.1)	94 (97.9)	96		
No response	*28 (18.4)*	*124 (81.6)*	*152*		
**Parity**					
1	1 (0.9)	112 (99.1)	113	1.643	†0.723
2	2 (1.3)	153 (98.7)	155		
3	3 (2.7)	108 (97.3)	111		
4	1 (1.9)	52 (98.1)	53		
≥5	0 (0)	11 (100)	11		
No response	*54 (26.1)*	*153 (73.9)*	*207*		
**History of child death**					
Yes	2 (3.8)	50 (96.2)	52	2.039	†0.214
No	59 (9.9)	539 (90.1)	598		
**No of living children**					
1	30 (18.)	131 (81.4)	161	37.399	†<0.001*
2	4 (2.3)	173 (97.7)	177		
3	6 (4.6)	125 (95.4)	131		
4	2 (4.0)	48 (96.0)	50		
≥5	0 (0)	13 (100)	13		
**Last delivery (months)**					
<6	22 (14.3)	132 (85.7)	154	13.777	0.008*
6-11	16 (9.5)	153 (90.5)	169		
12-17	7 (5.3)	125 (94.7)	132		
18-24	6 (3.9)	149 (96.1)	155		
No response	*10 (25.0)*	*30 (75.0)*	*40*		
**Outcome of last pregnancy**					
Live birth	61 (9.4)	587 (90.6)	648	0.208	†1.000
Still birth	0 (0)	2 (100)	2		

* Statistically significant † Fisher exact p
